# Microglial complex I activity: a crucial factor for smoldering inflammation in the central nervous system

**DOI:** 10.1038/s41392-024-01894-1

**Published:** 2024-07-31

**Authors:** Jana K. Sonner, Christina Mayer, Manuel A. Friese

**Affiliations:** Institute of Neuroimmunology and Multiple Sclerosis, University Medical Center Hamburg-Eppendorf, Hamburg, Germany

**Keywords:** Neuroimmunology, Neurological disorders

In their article published in *Nature*, Peruzotti-Jametta et al.^1^ identify metabolic reprogramming of microglia as a defining characteristic of their persistent and harmful activation during inflammatory neurodegeneration, which is observable in conditions like multiple sclerosis (MS). Subsequently, the authors demonstrate how mitigating mitochondrial dysregulation in microglia alleviates disease progression in an animal model of MS, thereby unveiling a potential novel target for the treatment of MS (Fig. 1).

**Fig. 1:**
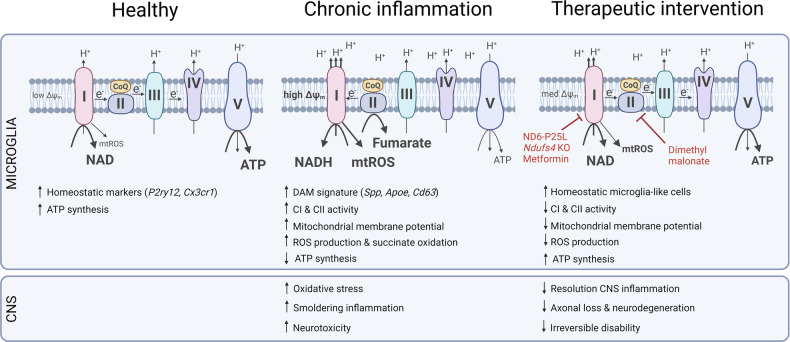
Mitochondrial complex I activity in disease-associated microglia perpetuates chronic neuroinflammation. During chronic inflammation, microglia repurpose mitochondria to generate reactive oxygen species (ROS) and cause oxidative stress within the central nervous system (CNS). Persistent low-grade inflammation induces neurotoxicity and clinical disability. Interference with complex I (CI) or complex II (CII) activity can normalize mitochondrial respiration and thereby halt persistent neuroinflammation. ATP adenosine triphosphate, CoQ coenzyme Q, DAM disease-associated microglia, NAD nicotinamide adenine dinucleotide, mtROS mitochondrial reactive oxygen species. Created with BioRender.com

Progressive MS is a disease that affects the entire central nervous system (CNS), including the brain and spinal cord. It is characterized by persistent low-grade inflammation causing axonal damage, widespread neuronal loss, and demyelination, independent of acute relapses.^2,3^ One hallmark of this low-grade inflammation, also called smoldering inflammation, is the continuous activation of myeloid cells in non-lesioned gray and white matter of the CNS, as well as in slowly expanding lesions. Myeloid cells accumulate at the lesion rim and release neurotoxic molecules such as pro-inflammatory cytokines and reactive oxygen species (ROS). Notably, the presence of these chronic active lesions, as can be detected by magnetic resonance imaging (MRI) is one of the best predictors of disease progression in people with MS (pwMS).^3^ However, our understanding of how myeloid cell activation contributes to smoldering inflammation is incomplete and to date no disease-modifying therapy has proven effective in the treatment of progressive MS.

Peruzotti-Jametta et al. tackled this challenge by dissecting the molecular signature of disease-associated microglia (DAM) in experimental autoimmune encephalomyelitis (EAE), an animal model of chronic CNS inflammation with MS-like pathology. To selectively isolate yolk sac-derived microglia and CNS-associated macrophages, as well as hematopoietic stem cell-derived infiltrating myeloid cells the authors utilize *Cx3cr1-YFP*^*creERT2*^*R26*^*tdTomato*^ fate-mapping mice^4^ to distinguish CNS-resident microglia from infiltrating myeloid cells. While earlier studies analyzed the composition and molecular signatures of myeloid cells primarily within the early and acute phases of the disease,^4^ the authors of the current study instead focused on the chronic phase (50 days after immunization) and aimed to decipher how microglia contribute to smoldering inflammation and neurotoxicity. Using single-cell RNA sequencing (scRNA-seq) of myeloid cells from healthy, acute and chronic EAE mice, the authors identified a distinct cluster of DAMs that increased in frequency during disease progression. Within this persistent DAM cluster, a subpopulation showed an enrichment of genes that are involved in mitochondrial complex I (CI) function, as well as other components of the mitochondrial respiratory chain. These findings were corroborated by immunohistochemical analyses both in EAE mice and post-mortem CNS tissue from secondary progressive MS patients. In these samples, microglia positive for the DAM-associated protein SPP1 and the CI-associated protein NDUFS4 were located both at the rim and within chronic active lesions. Furthermore, the authors re-analyzed two existing single-nuclei RNA sequencing (snRNA-seq) datasets from pwMS and control individuals. They identified a cluster of human microglia with a transcriptional profile comparable to their DAM cluster in EAE mice, providing further evidence for the potential relevance of these myeloid cells in sustaining smoldering inflammation.

In line with a previous study-which showed that an inflammatory challenge of macrophages leads to a shift from oxidative phosphorylation and ATP synthesis to reverse electron transport (RET) along the respiratory chain, resulting in ROS production^5^-Peruzzotti-Jametta et al. confirmed an increased mitochondrial membrane potential, elevated CI and complex II (CII) activity, as well as ROS generation in ex vivo isolated microglia from chronic EAE mice.

To mechanistically dissect the impact of an aberrant mitochondrial function, characterized by increased CI and CII activity in microglia, on neurotoxicity, the authors employed a variety of genetic and pharmacological interventions. Introducing a point mutation in the mitochondrial DNA (mtDNA) gene *Nd6*, a subunit of complex I, was sufficient to prevent microglial ROS production and neurite toxicity in vitro despite the induction of RET. In line with this, *Nd6* mice that carry the respective point mutation, showed an ameliorated EAE disease course accompanied by a normalization of mitochondrial respiration and reduced oxidative stress in microglia. In a sophisticated approach that specifically targeted microglial CI activity during the chronic phase of the disease, the authors assessed the therapeutic potential for chronic inflammatory disorders of the CNS. They induced a myeloid cell-specific knockout of the CI component *Ndufs4* by tamoxifen administration after the onset of EAE disease. Conditional *Ndufs4* knockout mice showed a remarkable decrease in disease activity during the chronic phase, a relative expansion of homeostatic microglia-like cells, lower expression of CI and CII in DAMs, and reduced neurodegeneration. Importantly, by utilizing their findings in a therapeutic approach, the administration of a combination of the CI inhibitor metformin and the CII inhibitor dimethyl malonate in EAE phenocopied key characteristics of the genetic interventions and reduced axonal loss. Together this study highlights the impact of dysregulated mitochondrial function on chronic CNS inflammation that drives neurodegeneration. Given that aberrant microglial activity is a hallmark not only of MS but also of other neuroinflammatory and neurodegenerative diseases, limiting CI activity through pharmacological interventions could be beneficial for a broader spectrum of CNS disorders.

Despite the plethora of approved therapeutics for patients with relapsing-remitting MS, treatment approaches for people with progressive MS are still limited. While small-molecule and antibody-based therapies can readily suppress the occurrence of relapses by targeting peripheral lymphocytes, they mostly fail to halt the progression of persistent inflammation within the CNS parenchyma.^2^ With myeloid cells being one of the predominant cell types within chronic active inflammatory MS lesions, they appear to be a suitable target to alleviate the inflammatory burden and thereby prevent neurodegeneration and irreversible disability. Yet, strategies to revert the pro-inflammatory phenotype of microglia are still lacking, and hopes for the highly anticipated Bruton’s tyrosine kinase (BTK) inhibitors have recently been dampened by initial reports of limited benefits in preventing relapses compared to existing therapies. With their study, Peruzzotti-Jametta et al. provide mechanistic insights into how aberrant mitochondrial CI activity drives oxidative stress and neurotoxicity in chronic CNS inflammation. Their data are backed by analyses in human specimens and thereby provide potential for clinical translatability. However, a potential limitation of the study is that the application of these findings to human disease is currently uncertain, as the chronic EAE model used by the authors only partially reflects the pathophysiology of slowly expanding lesions observed in progressive MS patients. Nevertheless, this study raises the expectations that ongoing clinical trials with the CI inhibitor metformin in MS patients may be beneficial in relieving the burden of chronic inflammatory disease activity.

## References

[1] Peruzzotti-Jametti, L. et al. Mitochondrial complex I activity in microglia sustains neuroinflammation. *Nature* **628**, 195–203 (2024). 38480879 10.1038/s41586-024-07167-9 PMC10990929

[2] Attfield, K. E., Jensen, L. T., Kaufmann, M., Friese, M. A. & Fugger, L. The immunology of multiple sclerosis. *Nat. Rev. Immunol.* **22**, 734–750 (2022). 35508809 10.1038/s41577-022-00718-z

[3] Woo, M. S., Engler, J. B. & Friese, M. A. The neuropathobiology of multiple sclerosis. *Nat. Rev. Neurosci*. 10.1038/s41583-024-00823-z (2024). 10.1038/s41583-024-00823-z 38789516

[4] Jordão, M. J. C. et al. Single-cell profiling identifies myeloid cell subsets with distinct fates during neuroinflammation. *Science* **363**, eaat7554 (2019). 30679343 10.1126/science.aat7554

[5] Mills, E. L. et al. Succinate dehydrogenase supports metabolic repurposing of mitochondria to drive inflammatory macrophages. *Cell* **167**, 457–470.e13 (2016). 27667687 10.1016/j.cell.2016.08.064 PMC5863951

